# Determinants of Physical Activity in the Cardiac Population: Protocol for a Systematic Review

**DOI:** 10.2196/39188

**Published:** 2022-09-07

**Authors:** Johanna Gutenberg, Stefan Tino Kulnik, Josef Niebauer, Rik Crutzen

**Affiliations:** 1 Ludwig Boltzmann Institute for Digital Health and Prevention Salzburg Austria; 2 Department of Health Promotion Care and Public Health Research Institute Maastricht University Maastricht Netherlands; 3 University Institute of Sports Medicine, Prevention and Rehabilitation and Research Institute of Molecular Sports Medicine and Rehabilitation Paracelsus Medical University Salzburg Austria

**Keywords:** behavior change, cardiac rehabilitation, cardiovascular disease, determinants, heart healthy, physical activity, protocol, secondary prevention, systematic review, cardiac disease, heart disease, clinician, health care worker, health intervention, decision-making, health promotion

## Abstract

**Background:**

Lack of physical activity is a critical contributing risk factor to cardiovascular disease. Hence, regular physical activity is a mainstay in the primary and secondary prevention of cardiovascular disease. Despite the extensive promotion of physical activity in both primary and secondary prevention programs, including cardiac rehabilitation, physical activity levels in the cardiac population remain low. Therefore, it is crucial to understand critical determinants that influence physical activity behavior.

**Objective:**

This study aims to deliver a systematic review of studies with collated observational data exploring the association between determinants and physical activity behavior in the target population. These new insights inform the design of future interventions targeted at lasting heart-healthy physical activity behavior in the cardiac population.

**Methods:**

Primary studies with observational quantitative data on determinants and their association with physical activity behavior in the cardiac population will be included. Information on relevant primary studies will be retrieved from various databases, including Embase, CINAHL, MEDLINE, PsycInfo, and Web of Science Core Collection. Six reviewers will independently double-screen articles. Studies will be selected according to the prespecified inclusion and exclusion criteria. Data will be extracted and entered into suitable worksheets. The US-based National Heart, Lung, and Blood Institute’s Study Quality Assessment Tool for Observational Studies will be used to assess the quality of all eligible primary studies. The results will be presented in a descriptive and narrative synthesis. If the type and quality of data are suitable, meta-analyses will be conducted. Study reporting will follow the PRISMA (Preferred Reporting Items for Systematic Reviews and Meta-Analyses) statement.

**Results:**

Data collection started in September 2020, and the literature search was updated in July 2021. Data synthesis is ongoing, and the literature search will be updated in October 2022.

**Conclusions:**

This review will be valuable to relevant stakeholders, including clinicians and health care professionals, intervention developers, and decision makers in health care. It lays a comprehensive foundation for understanding the determinants of physical activity to inform the design of secondary prevention interventions relevant to the cardiac population.

**Trial Registration:**

PROSPERO CRD42020206637; https://www.crd.york.ac.uk/prospero/display_record.php?RecordID=206637

**International Registered Report Identifier (IRRID):**

RR1-10.2196/39188

## Introduction

### Background

Cardiovascular disease (CVD) is the number one cause of death and disability worldwide, taking approximately 17.9 million lives each year [1]. The pre-eminence of cardiac events has not substantially changed over the past three decades [2]. This applies especially to the population older than 60 years. Numerous modifiable risk factors associated with an unhealthy lifestyle contribute to CVD, such as diabetes, hyperlipidemia, arterial hypertension, obesity, smoking, and physical inactivity. In a recent large-scale epidemiological study of over 155,000 participants across 21 high-, middle-, and low-income countries, it was estimated that 70% of CVD cases and deaths in the overall study population could be attributed to modifiable risk factors [3]. Although physical inactivity is one of the most deleterious cardiovascular risk factors, which co-influences other heart-critical risk factors such as dyslipidemia, diabetes mellitus type 2, obesity, and arterial hypertension, levels of physical activity remain well below current recommendations [4,5].

The current World Health Organization recommendations call for 150 to 300 minutes of moderate-intensity physical activity, 75 to 150 minutes of vigorous-intensity physical activity, or a combination of both for healthy adults per week. These recommendations are also a target for cardiac rehabilitation; nonetheless, a study surveying 8261 coronary patients from 27 European countries between 6 and 24 months after a cardiac event found that only 35% of respondents reported that they were performing planned physical activity to increase physical fitness, while 42% reported that they were not performing any planned physical activity and that they had no intention to do so [6].

In secondary prevention, cardiac rehabilitation programs promote physical activity for people with CVD. The concept of cardiac rehabilitation refers to interventions targeted at mitigating the effects of underlying heart conditions and restoring physiological, psychological, and societal functioning, reducing the overall risk of morbidity and mortality [7,8]. Although research on exercise-based cardiac rehabilitation offers good evidence of its positive effects on the physical and mental health of people with cardiovascular conditions, challenges remain in maintaining physical activity behavior after completing cardiac rehabilitation [9].

Reasons for nonmaintenance are multifactorial. Previous studies suggest that several determinants influence physical activity behavior in the cardiac population, namely, health beliefs [10-12], illness cognition [13-15], health literacy [16,17], sociodemographics [18,19], and health conditions and comorbidities [20-22]. While there have been individual observational studies and randomized controlled trials that have captured quantitative data on determinants of physical activity in the CVD population, to our knowledge to date, there have not been any systematic reviews and meta-analyses of these quantified associations between determinants and physical activity behavior. There is, therefore, an opportunity for the systematic collection and analysis of this body of data using a systematic review study design, providing collated insights into the determinants of physical activity and the strength of their associations with physical activity in this population.

### Rationale

No existing systematic and comprehensive collation and appraisal of the available data on the quantified association of different determinants with physical activity behavior in the cardiac population could be identified. It is expected that such findings may allow for a comparison of the relative importance of determinants for heart-healthy physical activity levels in people with CVD. (The lay term *heart-healthy* in this context is chosen deliberately to convey that the physical activity is intended to contribute to maintaining or improving cardiovascular health.) These findings can inform research and interventions concerned with health behavior change by shedding new light on how determinants operate within specific sociodemographic, social-cognitive, health-related, and physical environmental contexts.

### Objectives

The objectives of this systematic review are to:

- Identify relevant determinants of physical activity behavior in the cardiac population
- Determine the strength of the association between the identified determinants and physical activity behavior

## Methods

### Overview

This systematic review protocol specifies the conduct and reporting of a systematic review in compliance with the PRISMA (Preferred Reporting Items for Systematic Reviews and Meta-Analyses) statement. This protocol follows the 2015 PRISMA-P (Preferred Reporting Items for Systematic Review and Meta-Analysis Protocols) checklist [23] (Multimedia Appendix 1). This systematic review will include studies that provide observational data quantifying the association between determinants and the outcome of interest (ie, physical activity–related behavior) such as odds ratios, correlation coefficients, and multivariate regression coefficients. This could include studies with cross-sectional or longitudinal observational designs or secondary analyses of experimental studies (eg, analysis of data from a *usual care* control group). This protocol has been registered with PROSPERO (registration CRD42020206637).

### Eligibility Criteria

#### Search Domains

The search domains consist of population, determinants of physical activity, and outcome behavior (physical activity).

#### Study Populations

This systematic review focuses on ongoing self-initiated heart-healthy physical activity behavior of the adult cardiac population. The study population is defined as adults (aged ≥18 years) who fulfill the medical indications for cardiac rehabilitation. The reason for defining this population description is that a medical indication for cardiac rehabilitation implies an evidence-based recommendation for the person to carry out ongoing heart-healthy physical activity and exercise as part of their individual lifelong secondary CVD risk prevention.

The specific medical indications for cardiac rehabilitation vary between countries but commonly include, among others [4,24,25]:

- Acute coronary syndrome, including ST-elevation myocardial infarction and non–ST-elevation myocardial infarction
- Bypass surgery
- Other heart surgeries
- Heart and lung transplantation
- Chronic heart failure
- Percutaneous coronary intervention
- Chronic coronary heart disease
- Pulmonary hypertension
- Condition after an electrophysiological intervention
- Cardiac pacemaker or a defibrillator
- Hemodynamically stable arrhythmia

#### Determinants

Determinants (ie, factors that are associated with physical activity behavior) include socioeconomic, environmental, and psychological factors, as well as personal characteristics and demographics. The domain *determinants* includes search terms such as determinants, factors, influences, barriers, obstacles, facilitators, mediators, enablers, causes, reasons, triggers, contributors, predictors, and correlates. This systematic review will include both modifiable (eg, psychological factors) and nonmodifiable determinants (eg, demographics). However, the focus will lie on potentially modifiable determinants that are amenable to change, as the findings from this review should ideally inform future intervention designs to address these determinants. Examples for expected determinants under each determinant category are age, gender, and marital status in the demographics category; anxiety, depression, and kinesiophobia in the psychological factors category; self-efficacy, subjective norm, and illness perception in the cognitive factors category; and severity of cardiac condition and level of comorbidity in the morbidity category.

#### Outcome Behavior

Relevant for this systematic review will be any outcome that demonstrates physical activity behavior. Physical activity is defined as any bodily movement produced by skeletal muscles that results in energy expenditure at work or during leisure time such as sports, conditioning exercises, household tasks, and other activities [26]. Both self-reported and tracked physical activity data will be included. *Self-reported* physical activity data refers to information provided by self-completion of a questionnaire or by answering questions posed by a researcher or health care professional. *Tracked* refers to physical activity data that is collected through sensor devices, including consumer-grade devices (smartphones, smartwatches, pedometers, wearable activity trackers such as Fitbit) and research-grade sensors (inertial measurement units, accelerometers).

Different outcome measures will be considered, including measures describing the level of physical activity (eg, daily/weekly minutes of moderate and vigorous physical activity, step count, energy expenditure, and instrument-specific activity scores or categorization to activity levels) as well as descriptions of adherence to physical activity recommendations (eg, adherence to the World Health Organization physical activity recommendations).

Descriptions of dynamic subbehaviors (change) of physical activity over time (starting, stopping, increasing, reducing, and switching the type of activity, sport, or exercise) will be included, with the intention to identify determinants of change dynamics. More differentiated aspects of physical activity behavior over time are:

- Maintaining/sustaining
- Starting/beginning
- Changing frequency/intensity/time (duration)/type of physical activity (Frequency, Intensity, Time, and Type principle [27])
- Stopping/discontinuing

Explicitly excluded will be outcomes describing attendance at or completion of cardiac rehabilitation exercise programs and outcomes describing adherence to professionally guided home- or community-based exercise training interventions for people with CVD. Because these types of programs are generally highly resourced and time-limited, these outcomes are considered less helpful toward the aim of this review, which concerns ongoing self-initiated heart-healthy physical activity behavior.

#### Additional Criteria

This review includes publications in which precise association statistics such as odds ratios and correlation coefficients are presented. Publications that report on associations between determinants and outcome behavior without presenting precise association statistics will be excluded.

This review includes journal publications in English, Dutch, and German. To be able to apprehend state-of-the-art literature, the search includes journal publications from the past 15 years (2005-2020). Peer-reviewed literature only will be included to address the objectives specified above.

### Information Sources

Studies will be retrieved using five bibliographic databases, namely, MEDLINE via PubMed, Embase, Web of Science Core Collection, PsycINFO, and CINAHL. Preliminary searches of PROSPERO and the Cochrane Database of Systematic Reviews were performed to confirm that no systematic review with a similar research scope has previously been conducted or is currently underway, ensuring that this study delivers relevant and novel insights.

### Search Strategy

A scoping literature search of PubMed was performed by one researcher (STK) to identify which descriptors authors use for the population, determinants, and outcome behavior targeted by this study. From this, a detailed search strategy was developed for each electronic database by three researchers (JG, STK, RC) and an experienced librarian from Maastricht University Library to identify studies for inclusion. The complete search strategies for all bibliographic databases are provided in Multimedia Appendix 2. To reach a high level of methodological transparency and reproducibility, the search strategy will be piloted by two researchers (JG, STK) independently. The search strategy will be used for the identification of study records in relevant electronic databases.

The search strategy contains terms that refer to determinants and contributors associated with physical activity in cardiac rehabilitation and secondary prevention in people with cardiac disease; these terms will be adapted for use in all databases. The search strategy includes the main sets of keywords referring to “determinants,” “physical activity,” and “cardiac rehabilitation”.

The search terms were combined using the Boolean operators “AND” and “OR.” Subsequently, a search strategy was developed by combining Medical Subject Headings (MeSH) terms and word searches in the title or abstract. Publications were filtered by publication year range and human studies; no other filters were applied. The search strategy for the systematic review will be documented in a table stating the database, the date, the search terms, the database fields, and the number of search results.

### Data Management

Search results will be imported into the reference manager software EndNote (Clarivate). This facilitates systematic and comprehensive data management by storing, organizing, and merging duplicates of scholarly articles in one application. Titles and abstracts will then be transferred to a Google Sheet. The Google Sheet includes two worksheets. The first worksheet contains a queue with the inclusion criteria, drop-down menus, and a designed data-screening algorithm. When the screener chooses “No” in the drop-down function for any of the inclusion criteria, the final screening decision cell will automatically indicate “exclude”; if “unsure”, it switches to “?”; and if all inclusion criteria are fulfilled and can be answered with “Yes,” the final screening decision column automatically indicates “include.” The second working sheet consists of the working definitions for the three search domains “population,” “determinants,” and “outcome behaviour.” This Google Sheet design is intended to support and manage a larger group of reviewers by creating an efficient and convenient workflow for screening and data extraction, and to ensure that screening decisions and data extractions are documented and auditable.

### Study Selection Process

This review will include primary research studies only. The screening of records will be performed against the eligibility criteria. Disagreements between reviewers regarding screening decisions will be resolved by consensus or by a third independent reviewer’s decision.

The first phase of the study selection process consists of screening by title and abstract according to predefined dimensions: population, determinants, and outcome behavior. In phase two, the team will screen the full texts of documents to verify the studies’ eligibility. At each phase, independent double-screening will be conducted by a team of up to 8 reviewers. Discrepancies in screening decisions will be resolved by discussion, with the involvement of a third reviewer where necessary.

A flowchart following the PRISMA statement [23] will be created to document the selection process. The flowchart includes the search results, the removed primary studies after reviewing the title or abstract, the citations obtained in full text, the removed studies after full-text screening including duplicated records and irrelevant studies, and the ultimately included primary studies.

### Data Extraction

Data will be extracted by record number and include all relevant information: article ID, author, journal, study title, language, year of publication, study aim, study design, sample description (characteristics and size), setting and country, determinants, methods of assessing determinants, type of outcome, outcome assessment methods, types of effect sizes calculated for associations between determinants and outcome (odds ratios, correlation coefficients, multivariate regression coefficients), and reported effect sizes for association statistics with measures of precision (CIs, *R*^2^ values) and summary statistics for determinants and outcome parameters (frequencies, measures of central tendency, and spread). If associations have been reported for subgroups of the study sample, sample sizes of subsamples will be noted.

### Quality Assessment

To assess the methodological quality of included studies, the research team will use the US-based National Heart, Lung, and Blood Institute’s Study Quality Assessment Tool for Observational Studies [28]. This tool is suitable as it matches the chosen study designs and the level of detail required in the assessment, and it guides and enables the process of differentiating between internal validity (risk of bias) and external validity (generalizability). The quality of the individual studies will be assessed independently by two reviewers; disagreements will be resolved by discussion, and if necessary, a third reviewer will be consulted.

### Data Synthesis

An initial descriptive synthesis will be conducted using text and tables, including a summary of included studies, quality assessment, and a description of the risk of bias for individual studies. After that, a narrative synthesis will be conducted. The strength of associations between identified determinants and physical activity behavior will be assessed and described according to qualitative descriptors proposed by Rosenthal [29].

If the data is suitable, a quantitative synthesis (meta-analysis of effect sizes regarding determinants of physical activity) will be performed. Heterogeneity will be assessed using the *I*^2^ statistic and sensitivity analyses by comparison of subgroups stratified by clinical characteristics, outcome parameters, and study designs. The degree of heterogeneity observed will inform the decision to proceed to a meta-analysis and the choice of fixed-effects model (in case of homogeneous data) or random-effects model (in case of heterogeneous data). A forest plot will be constructed to graphically illustrate the meta-analysis. The possibility of publication bias will be examined using a funnel plot [30].

Depending on the types and numbers of primary studies identified, analyses of subsets may be performed as well, for example, according to a specific physical activity behavior such as stopping physical activity after cardiac rehabilitation.

### Amendments to the Protocol

All essential amendments to this protocol will be discussed and decided among the core study team (JG, STK, RC) and communicated among the wider team of reviewers. Amendments will be documented, specifying the timing and rationale for each amendment, added to the PROSPERO study entry, and reported in the final study report.

## Results

Data collection started in September 2020 and the literature search was updated in July 2021. Data synthesis is ongoing and the literature search will be updated in October 2022.

## Discussion

### Contribution to the Literature

To reach the objectives specified above, several methodological considerations and decisions have informed the protocol for this systematic review. One early principal decision in the review’s conceptualization was to search for data on determinants of physical activity behavior instead of searching for experimental evidence of behavior change interventions for physical activity in people with CVD. Both approaches are possible, depending on the objectives of interest. For example, systematic reviews and meta-analyses by Silva et al [31] and Bélanger-Gravel et al [32] collated experimental evidence of implementation intention interventions (eg, action planning) on physical activity behavior in mixed populations, including studies with healthy and general population samples as well as different clinical groups. These reviews were able to show small to moderate pooled effect sizes of intention interventions on physical activity, as eligibility criteria were a priori theoretically driven and included interventions based on the Theory of Self-Regulation and Theory of Planned Behaviour, resulting in the inclusion of theoretically coherent primary study interventions [31,32]. In this example of previous studies, the focus is on *interventions* targeting physical activity.

For physical activity behavior in the cardiac rehabilitation population, this approach may present limitations due to various and complex intervention concepts that have been trialed in the past, with differing and sometimes unspecified or poorly described underlying theoretical mechanisms of action. It is a recognized limitation in the literature on complex health care *interventions* that authors provide limited details on the underpinning theoretical approach, assumptions of mechanisms of action, and actual content and implementation of interventions [33,34]. For this reason, a decision was made to search for observational evidence of *determinants* of physical activity behavior in this target population, which relates more directly to understanding influences on behavior and is therefore likely to prove more informative for developing novel contextualized intervention concepts than experimental evidence of intervention effectiveness.

The systematic review and meta-analysis by Amireault et al [35] has a comparable focus area to this review and investigates the *determinants* of physical activity maintenance with potential long-term effectiveness to inform future interventions. However, its target population is healthy adults. Determinants of physical activity may be different for the cardiac rehabilitation population compared to healthy adults, as health conditions, comorbidities, and physiological factors such as kinesiophobia [36] may determine physical activity behavior differently in the cardiac rehabilitation population.

Another review with similar focus was published by Petter et al [37]. The authors collated evidence of social-ecological correlates of exercise in people with coronary heart disease and described 32 factors based on 121 included studies. The review by Petter et al [37] differs from this review in two key aspects. First, due to the lack of detailed reporting of association statistics, Petter et al [37] were unable to synthesize precise association statistics (as is the aim in this review) but interpreted the presence of associations across studies, that is, based on the number of studies supporting certain hypothesized associations (*detailed group procedure*). While this is a valid and useful approach, it is anticipated that our review will add value through synthesizing quantified associations from reported association statistics, thus enabling a more informed judgement on the relative importance of different determinants. Second, most studies included in the review by Petter et al [37] provided data on people’s exercise adherence during formal center-based and home-based cardiac rehabilitation, as opposed to sustained maintenance of heart-healthy physical activity outside of formal cardiac rehabilitation provision, which is the aim of this review.

In the definition of the study population, this review will focus on people with a medical indication for cardiac rehabilitation because this implies a recommendation for lifelong regular heart-healthy physical activity. It also indicates that this target population would (or should) enter formal clinical rehabilitation settings or have other encounters with cardiac clinical services (follow-up appointments, written communications), providing touchpoints at which theoretically and empirically informed behavior change interventions for sustainable healthy habit formation could ideally be leveraged. In the finalized search strategy, the search terms for relevant medical diagnoses were therefore combined with descriptors for cardiac rehabilitation, suitably contextualizing the search to the cardiac rehabilitation clinical pathway.

It was a deliberate decision, in the selection of the outcome for the systematic review, to focus on physical activity behavior as opposed to exercise capacity. Exercise capacity describes an individual’s physical fitness and constitutes a critical cardiac rehabilitation outcome. Typically, it is assessed through standardized ergometry [38] or other individual fitness tests such as the 6-minute walking test [39]. However, in this review, the focus lies on a more directly relatable outcome: habitual physical activity behavior in the target population, which also links to the official international recommendation for physical activity provided by the World Health Organization [40]. Moreover, the focus of interest in this review lies on individuals’ habitual physical activity behavior in the context of their everyday lives. Therefore, studies of attendance at or completion of cardiac rehabilitation exercise sessions will be excluded, as will studies of adherence to professionally guided home- or community-based exercise training interventions for people with CVD because these are generally highly resourced, time-limited, and not reflective of ongoing self-initiated heart-healthy physical activity behavior.

In defining the outcome of physical activity behavior, a deliberate decision was made to include both self-reported and tracked outcome data. It is often asserted that tracked (*objective*) physical activity measurements, for example, using accelerometers, pedometers, smartphones, or wearables, are preferable to self-reported (*subjective*) physical activity measures due to biases inherent in self-report methods, including misperception, recall bias, or social desirability bias [34,41]. For example, in a study of 1751 adults across ages 19 to 84, a comparison of self-reported (International Physical Activity Questionnaire) and tracked (ActiGraph accelerometer) physical activity over a 1-week period indicated that, on average, sedentary time was underreported and vigorous physical activity was overreported [42]. However, it may be helpful to view self-reported and tracked physical activity measures as two alternative or even complementary approaches, each with their advantages and disadvantages. Tracked physical activity measurements can be susceptible to technical failure or operator error, for example, when wearable devices are given out to study participants for more extended time periods and rely on participants for accurate application, handling, and recharging of the device. Other limitations of physical activity measurement devices include the inability to capture all types of activity automatically (in particular, swimming, cycling, and individualized exercise; eg, strength training can be difficult to detect), heterogeneity in device capabilities and data analytic processes, the burden of wear time for participants, and potential for high reactivity (ie, the impact of wearing a tracking device on the participant’s physical activity behavior) [43]. Advantages of self-reported physical activity measures are lower participant burden, cost, and time requirements of administration, particularly for one-off retrospective self-reporting of physical activity behavior (International Physical Activity Questionnaire [44]) as opposed to the daily completion of a physical activity diary or description of physical activity during *typical* time periods (Godin-Shephard Leisure-Time Physical Activity Questionnaire [45]).

### Limitations

Limitations of this systematic review are acknowledged. First, a date filter (2005 and later) is applied to the literature search. While this date filter is intended to identify studies more reflective of recent cardiac patient profiles under state-of-the-art medical and rehabilitation care, determinants of physical activity reported prior to 2005 may nevertheless be of relevance. Future research could extend the search parameters to include studies published prior to 2005 as well. Second, the results are restricted to peer-reviewed literature. This could lead to potential publication bias due to missing null results that are more likely to be disseminated in conference abstracts and gray literature than in peer-reviewed articles. Future research could address this limitation by widening eligibility criteria in addition to seeking access to unpublished data sets if necessary.

### Conclusions

Considering those beforementioned methodological aspects, this review will be valuable to relevant stakeholders, including clinicians and health care professionals, intervention developers, and decision makers in health care. It will shed new light on the relevance and significance of modifiable (eg, health belief, health literacy, illness cognition, or structural barriers for executing physical activity behavior) and nonmodifiable (eg, sociodemographics, education, health insurance, or income) determinants of physical activity to maintain physical activity behavior.

## Appendix

Multimedia Appendix 1 PRISMA-P (Preferred Reporting Items for Systematic Review and Meta-Analysis Protocols) 2015 checklist: recommended items to address in a systematic review protocol.

Multimedia Appendix 2 Search strategies for a systematic review on determinants of physical activity in the cardiac population.

## Abbreviations

CVD: cardiovascular disease
MeSH: Medical Subject Headings
PRISMA: Preferred Reporting Items for Systematic Reviews and Meta-Analyses
PRISMA-P: Preferred Reporting Items for Systematic Review and Meta-Analysis Protocols

## References

[1] (2021). Cardiovascular diseases (CVDs). World Health Organization.

[2] GBD 2019 Diseases and Injuries Collaborators (2020). Global burden of 369 diseases and injuries in 204 countries and territories, 1990-2019: a systematic analysis for the Global Burden of Disease Study 2019. Lancet.

[3] Yusuf S, Joseph P, Rangarajan S, Islam S, Mente A, Hystad P, Brauer M, Kutty VR, Gupta R, Wielgosz A, AlHabib KF, Dans A, Lopez-Jaramillo P, Avezum A, Lanas F, Oguz A, Kruger IM, Diaz R, Yusoff K, Mony P, Chifamba J, Yeates K, Kelishadi R, Yusufali A, Khatib R, Rahman O, Zatonska K, Iqbal R, Wei L, Bo H, Rosengren A, Kaur M, Mohan V, Lear SA, Teo KK, Leong D, O'Donnell M, McKee M, Dagenais G (2020). Modifiable risk factors, cardiovascular disease, and mortality in 155 722 individuals from 21 high-income, middle-income, and low-income countries (PURE): a prospective cohort study. Lancet.

[4] Pelliccia A, Sharma S, Gati S, Bäck M, Börjesson M, Caselli S, Collet J, Corrado D, Drezner JA, Halle M, Hansen D, Heidbuchel H, Myers J, Niebauer J, Papadakis M, Piepoli MF, Prescott E, Roos-Hesselink JW, Graham Stuart A, Taylor RS, Thompson PD, Tiberi M, Vanhees L, Wilhelm M, ESC Scientific Document Group (2021). 2020 ESC Guidelines on sports cardiology and exercise in patients with cardiovascular disease. Eur Heart J.

[5] Nystoriak MA, Bhatnagar A (2018). Cardiovascular effects and benefits of exercise. Front Cardiovasc Med.

[6] Kotseva K, De Backer G, De Bacquer D, Rydén L, Hoes A, Grobbee D, Maggioni A, Marques-Vidal P, Jennings C, Abreu A, Aguiar C, Badariene J, Bruthans J, Castro Conde A, Cifkova R, Crowley J, Davletov K, Deckers J, De Smedt D, De Sutter J, Dilic M, Dolzhenko M, Dzerve V, Erglis A, Fras Z, Gaita D, Gotcheva N, Heuschmann P, Hasan-Ali H, Jankowski P, Lalic N, Lehto S, Lovic D, Mancas S, Mellbin L, Milicic D, Mirrakhimov E, Oganov R, Pogosova N, Reiner Z, Stöerk S, Tokgözoğlu L, Tsioufis C, Vulic D, Wood D, EUROASPIRE Investigators* (2019). Lifestyle and impact on cardiovascular risk factor control in coronary patients across 27 countries: results from the European Society of Cardiology ESC-EORP EUROASPIRE V registry. Eur J Prev Cardiol.

[7] Taylor RS, Brown A, Ebrahim S, Jolliffe J, Noorani H, Rees K, Skidmore B, Stone JA, Thompson DR, Oldridge N (2004). Exercise-based rehabilitation for patients with coronary heart disease: systematic review and meta-analysis of randomized controlled trials. Am J Med.

[8] Anderson L, Taylor RS (2014). Cardiac rehabilitation for people with heart disease: an overview of Cochrane systematic reviews. Cochrane Database Syst Rev.

[9] Alves AJ, Viana JL, Cavalcante SL, Oliveira NL, Duarte JA, Mota J, Oliveira J, Ribeiro F (2016). Physical activity in primary and secondary prevention of cardiovascular disease: overview updated. World J Cardiol.

[10] Forestier C, Sarrazin P, Sniehotta F, Allenet B, Heuzé JP, Gauchet A, Chalabaev A (2020). Do compensatory health beliefs predict behavioural intention in a multiple health behaviour change context? Evidence in individuals with cardiovascular diseases?. Psychol Health Med.

[11] Webster R, Heeley E (2010). Perceptions of risk: understanding cardiovascular disease. Risk Manag Healthc Policy.

[12] Chiou A, Wang H, Chan P, Ding Y, Hsu K, Kao H (2009). Factors associated with behavior modification for cardiovascular risk factors in patients with coronary artery disease in northern Taiwan. J Nurs Res.

[13] Reges O, Vilchinsky N, Leibowitz M, Khaskia A, Mosseri M, Kark JD (2013). Illness cognition as a predictor of exercise habits and participation in cardiac prevention and rehabilitation programs after acute coronary syndrome. BMC Public Health.

[14] Steca P, Pancani L, Greco A, D'Addario M, Magrin ME, Miglioretti M, Sarini M, Scrignaro M, Vecchio L, Cesana F, Giannattasio C, Fattirolli F, Zanettini R (2015). Changes in dietary behavior among coronary and hypertensive patients: a longitudinal investigation using the health action process approach. Appl Psychol Health Well Being.

[15] Leventhal EA (1984). Aging and the perception of illness. Res Aging.

[16] Jennings C, De Bacquer D, Prescott E, Hansen T, Gale C, Astin F (2018). MS03.3 factors influencing patients’ self-reported lifestyle changes and medication adherence following an acute cardiac event in 12 countries: a specialist study within the Euroaspire V (EAV) survey. Global Heart.

[17] Safeer RS, Cooke CE, Keenan J (2006). The impact of health literacy on cardiovascular disease. Vasc Health Risk Manag.

[18] Daly J, Sindone AP, Thompson DR, Hancock K, Chang E, Davidson P (2002). Barriers to participation in and adherence to cardiac rehabilitation programs: a critical literature review. Prog Cardiovasc Nurs.

[19] Bergman P, Grjibovski AM, Hagströmer M, Bauman A, Sjöström M (2008). Adherence to physical activity recommendations and the influence of socio-demographic correlates - a population-based cross-sectional study. BMC Public Health.

[20] Lacombe J, Armstrong MEG, Wright FL, Foster C (2019). The impact of physical activity and an additional behavioural risk factor on cardiovascular disease, cancer and all-cause mortality: a systematic review. BMC Public Health.

[21] Kvaavik E, Batty GD, Ursin G, Huxley R, Gale CR (2010). Influence of individual and combined health behaviors on total and cause-specific mortality in men and women: the United Kingdom health and lifestyle survey. Arch Intern Med.

[22] Franco OH, de Laet C, Peeters A, Jonker J, Mackenbach J, Nusselder W (2005). Effects of physical activity on life expectancy with cardiovascular disease. Arch Intern Med.

[23] Moher D, Liberati A, Tetzlaff J, Altman DG, PRISMA Group (2009). Preferred reporting items for systematic reviews and meta-analyses: the PRISMA statement. PLoS Med.

[24] Balady GJ, Ades PA, Bittner VA, Franklin BA, Gordon NF, Thomas RJ, Tomaselli GF, Yancy CW, American Heart Association Science Advisory and Coordinating Committee (2011). Referral, enrollment, and delivery of cardiac rehabilitation/secondary prevention programs at clinical centers and beyond: a presidential advisory from the American Heart Association. Circulation.

[25] Niebauer J, Mayr K, Tschentscher M, Pokan R, Benzer W (2013). Outpatient cardiac rehabilitation: the Austrian model. Eur J Prev Cardiol.

[26] Caspersen CJ, Powell KE, Christenson GM (1985). Physical activity, exercise, and physical fitness: definitions and distinctions for health-related research. Public Health Rep.

[27] Burnet K, Kelsch E, Zieff G, Moore JB, Stoner L (2019). How fitting is F.I.T.T.?: A perspective on a transition from the sole use of frequency, intensity, time, and type in exercise prescription. Physiol Behav.

[28] (2014). Quality assessment tool for observational cohort and cross-sectional studies. National Heart, Lung, and Blood Institute.

[29] Rosenthal J (1996). Qualitative descriptors of strength of association and effect size. J Soc Serv Res.

[30] Mueller M, D'Addario M, Egger M, Cevallos M, Dekkers O, Mugglin C, Scott P (2018). Methods to systematically review and meta-analyse observational studies: a systematic scoping review of recommendations. BMC Med Res Methodol.

[31] Silva MAVD, São-João TM, Brizon VC, Franco DH, Mialhe FL (2018). Impact of implementation intentions on physical activity practice in adults: a systematic review and meta-analysis of randomized clinical trials. PLoS One.

[32] Bélanger-Gravel A, Godin G, Amireault S (2013). A meta-analytic review of the effect of implementation intentions on physical activity. Health Psychol Rev.

[33] O'Cathain A, Croot L, Duncan E, Rousseau N, Sworn K, Turner KM, Yardley L, Hoddinott P (2019). Guidance on how to develop complex interventions to improve health and healthcare. BMJ Open.

[34] Hoffmann TC, Glasziou PP, Boutron I, Milne R, Perera R, Moher D, Altman DG, Barbour V, Macdonald H, Johnston M, Lamb SE, Dixon-Woods M, McCulloch P, Wyatt JC, Chan A, Michie S (2014). Better reporting of interventions: template for intervention description and replication (TIDieR) checklist and guide. BMJ.

[35] Amireault S, Godin G, Vézina-Im L (2013). Determinants of physical activity maintenance: a systematic review and meta-analyses. Health Psychol Rev.

[36] Bäck M, Cider Å, Herlitz J, Lundberg M, Jansson B (2013). The impact on kinesiophobia (fear of movement) by clinical variables for patients with coronary artery disease. Int J Cardiol.

[37] Petter M, Blanchard C, Kemp KAR, Mazoff AS, Ferrier SN (2009). Correlates of exercise among coronary heart disease patients: review, implications and future directions. Eur J Cardiovasc Prev Rehabil.

[38] Reich B, Benzer W, Harpf H, Hofmann P, Mayr K, Ocenasek H, Podolsky A, Pokan R, Porodko M, Puelacher C, Sareban M, Traninger H, Ziegelmeyer W, Niebauer J (2020). Efficacy of extended, comprehensive outpatient cardiac rehabilitation on cardiovascular risk factors: a nationwide registry. Eur J Prev Cardiol.

[39] Bierbauer W, Scholz U, Bermudez T, Debeer D, Coch M, Fleisch-Silvestri R, Nacht C, Tschanz H, Schmid J, Hermann M (2020). Improvements in exercise capacity of older adults during cardiac rehabilitation. Eur J Prev Cardiol.

[40] Bull FC, Al-Ansari SS, Biddle S, Borodulin K, Buman MP, Cardon G, Carty C, Chaput J, Chastin S, Chou R, Dempsey PC, DiPietro L, Ekelund U, Firth J, Friedenreich CM, Garcia L, Gichu M, Jago R, Katzmarzyk PT, Lambert E, Leitzmann M, Milton K, Ortega FB, Ranasinghe C, Stamatakis E, Tiedemann A, Troiano RP, van der Ploeg HP, Wari V, Willumsen JF (2020). World Health Organization 2020 guidelines on physical activity and sedentary behaviour. Br J Sports Med.

[41] Meinhart F, Stütz T, Sareban M, Kulnik ST, Niebauer J (2020). Mobile technologies to promote physical activity during cardiac rehabilitation: a scoping review. Sensors (Basel).

[42] Dyrstad SM, Hansen BH, Holme IM, Anderssen SA (2014). Comparison of self-reported versus accelerometer-measured physical activity. Med Sci Sports Exerc.

[43] Silfee VJ, Haughton CF, Jake-Schoffman DE, Lopez-Cepero A, May CN, Sreedhara M, Rosal MC, Lemon SC (2018). Objective measurement of physical activity outcomes in lifestyle interventions among adults: a systematic review. Prev Med Rep.

[44] Hagströmer M, Patterson E International Physical Activity Questionnaire.

[45] Godin G (2011). The Godin-Shephard leisure-time physical activity questionnaire. Health Fitness J Canada.

