# Supplementary material for: Determinants of Physical Activity in the Cardiac Population: Protocol for a Systematic Review
Source: JMIR Res Protoc. 2022 Sep 7;11(9):e39188. doi: 10.2196/39188 (PMC9494216; doi:10.2196/39188)
Supplement: Multimedia Appendix 2 [file resprot_v11i9e39188_app2.docx]

## Appendix 2. Search strategies for a systematic review on determinants of physical activity in the cardiac population

**MEDLINE / PubMed**

| #1 | "Cardiac Rehabilitation"[Mesh] OR cardiac rehabilitation*[tiab] OR cardiovascular rehabilitation*[tiab] OR ((rehabilit*[tiab] OR "rehabilitation" [Subheading]) AND ("Cardiac Surgical Procedures"[Mesh] OR "Reperfusion"[Mesh] OR "Thoracic Surgery"[Mesh] OR "Stents"[Mesh] OR "Cardiopulmonary Bypass"[Mesh] OR arterial bypass*[tiab] OR artery bypass*[tiab] OR arterial switch*[tiab] OR cardiopulmonary bypass*[tiab] OR coronary bypass*[tiab] OR heart bypass*[tiab] OR heart massage*[tiab] OR heart transplantation*[tiab] OR heart valve prosthesis implantation*[tiab] OR pericardi*[tiab] OR reperfusion*[tiab] OR thoracic surgery[tiab] OR heart surgery[tiab] OR stent*[tiab] OR "Cardiovascular Diseases"[Mesh:NoExp] OR "Heart Diseases"[Mesh] OR "Hypertension"[Mesh] OR heart disease*[tiab] OR cardiac*[tiab] OR cardio*[tiab] OR Angina[tiab] OR endocardi*[tiab] OR heart aneurysm*[tiab] OR heart arrest*[tiab] OR heart defect*[tiab] OR heart failure*[tiab] OR heart neoplasm*[tiab] OR heart rhythm*[tiab] OR heart rupture*[tiab] OR heart valve disease*[tiab] OR hypertension*[tiab] OR myocardi*[tiab] OR ventricular dysfunction*[tiab] OR ventricular outflow obstruction*[tiab])) |
| --- | --- |
| #2 | "Motivation"[Mesh] OR "Self Care"[Mesh] OR "Self-Management"[Mesh] OR "Self Efficacy"[Mesh] OR "Personal Satisfaction"[Mesh] OR "Patient Compliance"[Mesh:NoExp] OR "Patient Participation"[Mesh] OR "Patient Satisfaction"[Mesh] OR "Personal Autonomy"[Mesh] OR "Self Concept"[Mesh] OR motivation[tiab] OR self care[tiab] OR self-management[tiab] OR self efficacy[tiab] OR personal satisfaction[tiab] OR patient compliance[tiab] OR patient participation[tiab] OR patient satisfaction[tiab] OR self concept[tiab] OR patient empower*[tiab] OR patient involve*[tiab] OR patient engage*[tiab] OR personal autonomy[tiab] OR factor*[ti] OR influenc*[ti] OR barrier*[ti] OR obstacle*[ti] OR facilit*[ti] OR mediator*[ti] OR enable*[ti] OR trigger*[ti] OR predict*[ti] OR correlat*[ti] OR determin*[ti] OR incentive*[ti] OR uptake[ti] OR motiv*[ti] OR engag*[ti] OR activation[tiab] OR self determination[tiab] OR rationali*[ti] OR reason*[ti] OR contribut*[ti] OR cause*[ti] OR adherence[ti] OR behavior[ti] OR behaviour[ti] OR characteristic*[ti] OR self-efficacy[ti] OR compliance[ti] OR attitude*[ti] |
| #3 | "Movement"[Mesh] OR "Health Behavior"[Mesh] OR "Health Promotion"[Mesh] OR "Sports"[Mesh] OR movement[tiab] OR physical activit*[tiab] OR routine*[tiab] OR sport*[tiab] OR training[tiab] OR physical exercis*[tiab] OR workout[tiab] OR motion[tiab] OR health monitoring[tiab] OR health behav*[tiab] OR health promotion*[tiab] OR motor activit*[tiab] OR health educat*[tiab] OR ((behaviour*[tiab] OR behavior*[tiab]) AND (modification*[tiab])) |
| #4 | #1 AND #2 AND #3 |
| #5 | NOT (Animals[Mesh] NOT Humans[Mesh]) |
| #6 | #4 AND #5 |
| #7 | Additional filters: publication year 2005-2020 |

**Embase**

| #1 | heart rehabilitation/ or cardiac rehabilitation*.ti,ab,kw. or cardiovascular rehabilitation*.ti,ab,kw. or ((rehabilit*.ti,ab,kw. or rh.fs) and (exp heart surgery/ or thorax surgery/ or thoracic aortic surgery/ or cardiovascular stent/ or exp reperfusion/ or coronary reperfusion/ or vascular surgery*.ti,ab,kw. or artery bypass*.ti,ab,kw. or arterial switch*.ti,ab,kw. or cardiopulmonary bypass*.ti,ab,kw. or coronary bypass*.ti,ab,kw. or heart bypass*.ti,ab,kw. or heart massage*.ti,ab,kw. or heart transplantation*.ti,ab,kw. or heart valve prosthesis implantation*.ti,ab,kw. or pericardi*.ti,ab,kw. or reperfusion*.ti,ab,kw. or thoracic surgery.ti,ab,kw. or heart surgery.ti,ab,kw. or stent*.ti,ab,kw. or exp cardiovascular disease/ or heart disease*.ti,ab,kw. or cardiac*.ti,ab,kw. or cardio*.ti,ab,kw. or Angina.ti,ab,kw. or endocardi*.ti,ab,kw. or heart aneurysm*.ti,ab,kw. or heart arrest*.ti,ab,kw. or heart defect*.ti,ab,kw. or heart failure*.ti,ab,kw. or heart neoplasm*.ti,ab,kw. or heart rhythm*.ti,ab,kw. or heart rupture*.ti,ab,kw. or heart valve disease*.ti,ab,kw. or hypertension*.ti,ab,kw. or myocardi*.ti,ab,kw. or ventricular dysfunction*.ti,ab,kw. or ventricular outflow obstruction*.ti,ab,kw.)) |
| --- | --- |
| #2 | Motivation/ OR exp self care/ OR exp self concept/ OR exp satisfaction/ OR patient compliance/ OR patient participation/ OR personal autonomy/ OR motivation.ti,ab,kw. OR self care.ti,ab,kw. OR self management.ti,ab,kw. OR self efficacy.ti,ab,kw. OR personal satisfaction.ti,ab,kw. OR patient compliance.ti,ab,kw. OR patient participation.ti,ab,kw. OR patient satisfaction.ti,ab,kw. OR self concept.ti,ab,kw. OR patient empower*.ti,ab,kw. OR patient involve*.ti,ab,kw. OR patient engage*.ti,ab,kw. OR personal autonomy.ti,ab,kw. OR factor*.ti. OR influenc*.ti. OR barrier*.ti. OR obstacle*.ti. OR facilit*.ti. OR mediator*.ti. OR enable*.ti. OR trigger*.ti. OR predict*.ti. OR correlat*.ti. OR determin*.ti. OR incentive*.ti. OR uptake.ti. OR motiv*.ti. OR engag*.ti. OR activation.ti,ab,kw. OR self determination.ti,ab,kw. OR rationali*.ti. OR reason*.ti. OR contribut*.ti. OR cause*.ti. OR adherence.ti. OR behavior.ti. OR behaviour.ti. OR characteristic*.ti. OR self-efficacy.ti. OR compliance.ti. OR attitude*.ti. |
| #3 | exp"movement (physiology)"/ OR exp health behavior/ OR health promotion/ OR exp "sports and sport related phenomena"/ OR movement.ti,ab,kw. OR physical activit*.ti,ab,kw. OR routine*.ti,ab,kw. OR sport*.ti,ab,kw. OR training.ti,ab,kw. OR workout.ti,ab,kw. OR motion.ti,ab,kw. OR leisure activit*.ti,ab,kw. OR physical exercis*.ti,ab,kw. OR health monitoring.ti,ab,kw. OR health behav*.ti,ab,kw. OR health promotion*.ti,ab,kw. OR motor activit*.ti,ab,kw. OR health educat*.ti,ab,kw. OR ((behaviour*.ti,ab,kw. OR behavior*.ti,ab,kw.) AND (modification*.ti,ab,kw.)) |
| #4 | #1 AND #2 AND #3 |
| #5 | Filters: Journal Article, from 2005 – 2020 |
| #6 | NOT ((exp animal/ or nonhuman/) NOT exp human/) |

**Web of Science Core Collection**

| #1 | (TS=rehabilit*) AND TS=(arterial bypass* OR artery bypass* OR arterial switch* OR cardiopulmonary bypass* OR coronary bypass* OR heart bypass* OR heart massage* OR heart transplantation* OR heart valve prosthesis implantation* OR pericardi* OR reperfusion* OR thoracic surgery OR heart surgery OR stent* OR heart disease* OR cardiac* OR cardio* OR Angina OR endocardi* OR heart aneurysm* OR heart arrest* OR heart defect* OR heart failure* OR heart neoplasm* OR heart rhythm* OR heart rupture* OR heart valve disease* OR hypertension* OR myocardi* OR ventricular dysfunction* OR ventricular outflow obstruction*) |
| --- | --- |
| #2 | TS=(self care OR self-management OR self efficacy OR personal satisfaction OR patient compliance OR patient participation OR patient satisfaction OR self concept OR patient empower* OR patient involve* OR patient engage* OR personal autonomy) OR TI=(factor* OR influenc* OR barrier* OR obstacle* OR facilit* OR mediator* OR enable* OR trigger* OR predict* OR correlat* OR determin* OR incentive* OR uptake OR motiv* OR engag* OR activation OR self determination OR rationali* OR reason* OR contribut* OR cause* OR adherence OR behavior OR behaviour OR characteristic* OR self-efficacy OR compliance OR attitude*) |
| #3 | TS=(movement OR physical activit* OR routine* OR sport* OR training OR workout OR motion OR physical exercis* OR health monitoring OR health behav* OR health promotion* OR motor activit* OR health educat*) OR TS=((behaviour* OR behavior*) AND modification*) |
| #4 | #1 AND #2 AND #3 |
| #5 | Filters: 2005 – 2020 |

**PsycINFO**

| #1 | DE "Rehabilitation" OR AB cardiac rehabilitation* OR TI cardiac rehabilitation* OR AB cardiovascular rehabilitation* OR TI cardiovascular rehabilitation* OR ((AB rehabilit* OR TI rehabilit*) AND (DE "Heart Surgery" OR MA thorax surgery OR MA stent OR MA Cardiopulmonary Bypass OR AB arterial bypass* OR TI arterial bypass* OR AB artery bypass* OR TI artery bypass* OR AB arterial switch* OR TI arterial switch* OR AB cardiopulmonary bypass* OR TI cardiopulmonary bypass* OR AB coronary bypass* OR TI coronary bypass* OR AB heart bypass* OR TI heart bypass* OR AB heart massage* OR TI heart massage* OR AB heart transplantation* OR TI heart transplantation* OR AB heart valve prosthesis implantation* OR TI heart valve prosthesis implantation* OR AB pericardi* OR TI pericardi* OR AB reperfusion* OR TI reperfusion* OR AB thoracic surgery OR TI thoracic surgery OR AB heart surgery OR TI heart surgery OR AB stent* OR TI stent* OR DE "Cardiovascular Disorders" OR DE "Cerebrovascular Disorders" OR DE "Heart Disorders" OR DE "Hypertension" OR heart disease* OR TI heart disease* OR cardiac* OR TI cardiac* OR AB cardio* OR TI cardio* OR AB Angina OR TI Angina OR AB endocardi* OR TI endocardi* OR AB heart aneurysm* OR TI heart aneurysm* OR AB heart arrest* OR TI heart arrest* OR AB heart defect* OR TI heart defect* OR AB heart failure* OR TI heart failure* OR AB heart neoplasm* OR TI heart neoplasm* OR AB heart rhythm* OR TI heart rhythm* OR AB heart rupture* OR TI heart rupture* OR AB heart valve disease* OR TI heart valve disease* OR AB hypertension* OR TI hypertension* OR AB myocardi* OR TI myocardi* OR AB ventricular dysfunction* OR TI ventricular dysfunction* OR AB ventricular outflow obstruction* OR TI ventricular outflow obstruction*)) |
| --- | --- |
| #2 | DE "Motivation" OR DE "Self-Care" OR DE "Self-Management" OR DE "Self-Efficacy" OR MA Personal Satisfaction OR MA Patient Compliance OR DE "Client Participation" OR MA Patient Satisfaction OR DE "Autonomy" OR DE "Self-Concept" OR TI self care OR TI self-management OR TI self efficacy OR TI personal satisfaction OR TI patient compliance OR TI patient participation OR TI patient satisfaction OR TI Self Concept OR TI patient empower* OR TI patient involve* OR TI patient engage* OR TI personal autonomy OR AB self care OR AB self-management OR AB self efficacy OR AB personal satisfaction OR AB patient compliance OR AB patient participation OR AB patient satisfaction OR AB Self Concept OR AB patient empower* OR AB patient involve* OR AB patient engage* OR AB personal autonomy TI factor* OR TI influenc* OR TI barrier* OR TI obstacle*OR TI facilit* OR TI mediator* OR TI enable* OR TI trigger* OR TI predict* OR TI correlat* OR TI determin* OR TI incentive* OR TI uptake OR TI motiv* OR TI engag* OR TI activation OR TI self determination OR TI rationali* ORTI reason* OR TI contribut* OR TI cause* OR TI adherence OR TI behavior OR TI behaviour OR TI characteristic* OR TI self-efficacy OR TI compliance OR TI attitude* |
| #3 | MA Movement OR DE "Health Behavior" OR DE "Sports" OR MA Health Promotion OR TI movement OR TI physical activit* OR TI routine* OR TI sport* OR TI training OR TI workout OR TI motion OR TI health monitoring OR TI health behav* OR TI health promotion* OR TI motor activit* OR TI health educat* OR ((TI behaviour* OR TI behavior*) AND (TI modification*)) OR TI movement OR AB physical activit* OR AB routine* OR AB sport* OR AB training OR AB workout OR AB motion OR AB health monitoring OR AB health behav* OR AB health promotion* OR AB motor activit* OR AB health educat* OR ((TI behaviour* OR AB behavior*) AND (TI modification*)) |
| #4 | #1 AND #2 AND #3 |
| #5 | Filters: from 2005 – 2020 |

**CINAHL**

| #1 | (MH "Rehabilitation, Cardiac+") OR AB(cardiac rehabilitation*) OR TI(cardiac rehabilitation*) OR AB(cardiovascular rehabilitation*) OR TI(cardiovascular rehabilitation*) OR ((AB(rehabilit*) OR TI(rehabilit*) OR (MW "RH")) AND ((MH "Surgery, Cardiovascular+") OR (MH "Thoracic Surgery") OR (MH "Heart Surgery+") OR (MH "Myocardial Reperfusion") OR AB(arterial bypass*) OR TI(arterial bypass*) OR AB(artery bypass*) OR TI(artery bypass*) OR AB(arterial switch*) OR TI(arterial switch*) OR AB(cardiopulmonary bypass*) OR TI(cardiopulmonary bypass*) OR AB(coronary bypass*) OR TI(coronary bypass*) OR AB(heart bypass*) OR TI(heart bypass*) OR AB(heart massage*) OR TI(heart massage*) OR AB(heart transplantation*) OR TI(heart transplantation*) OR AB(heart valve prosthesis implantation*) OR TI(heart valve prosthesis implantation*) OR AB(pericardi*) OR TI(pericardi*) OR AB(reperfusion*) OR TI(reperfusion*) OR AB(thoracic surgery) OR TI(thoracic surgery) OR AB(heart surgery) OR TI(heart surgery) OR AB(stent*) OR TI(stent*) OR (MH "Cardiovascular Diseases") OR (MH "Heart Diseases+") OR (MH "Hypertension+") OR AB(heart disease*) OR TI(heart disease*) OR AB(cardiac*) OR TI(cardiac*) OR AB(cardio*) OR TI(cardio*) OR AB(Angina”) OR TI(Angina”) OR AB(endocardi*) OR TI(endocardi*) OR AB(heart aneurysm*) OR TI(heart aneurysm*) OR AB(heart arrest*) OR TI(heart arrest*) OR AB(heart defect*) OR TI(heart defect*) OR AB(heart failure*) OR TI(heart failure*) OR AB(heart neoplasm*) OR TI(heart neoplasm*) OR AB(heart rhythm*) OR TI(heart rhythm*) OR AB(heart rupture*) OR TI(heart rupture*) OR AB(heart valve disease*) OR TI(heart valve disease*) OR AB(hypertension*) OR TI(hypertension*) OR AB(myocardi*) OR TI(myocardi*) OR AB(ventricular dysfunction*) OR TI (“ventricular dysfunction*) OR AB(ventricular outflow obstruction*) OR TI(ventricular outflow obstruction*”))) |
| --- | --- |
| #2 | (MH "Motivation+") OR (MH "Self Care") OR (MH "Self-Management") OR (MH "Self-Efficacy") OR (MH "Personal Satisfaction") OR (MH "Patient Compliance") OR (MH "Consumer Participation") OR (MH "Patient Autonomy") OR AB(motivation) OR TI (motivation) OR AB(self care”) OR TI(self care”) OR AB(self-management) OR TI(self-management) OR AB(self efficacy) OR TI(self efficacy) OR AB(personal satisfaction) OR TI(personal satisfaction) OR AB(patient compliance) OR TI(patient compliance) OR AB(patient participation) OR TI(patient participation) OR AB(patient satisfaction) TI(patient satisfaction) OR AB(Self Concept) OR TI(Self Concept) OR AB(patient empower*) OR TI(patient empower*) OR AB(patient involve*) OR TI(patient involve*) OR AB(patient engage*) OR TI(patient engage*) OR AB(personal autonomy) OR TI(personal autonomy) OR TI(factor*) OR TI(influenc*) OR TI(barrier*) OR TI(obstacle*) OR TI(facilit*) OR TI(mediator*) OR TI(enable*) OR TI(trigger*) OR TI(predict*) OR TI(correlat*) OR TI(determin*) OR TI(incentive*) OR TI(uptake) OR TI(motiv*) OR TI(engag*) OR TI(activation) OR TI(self determination) OR TI(rationali*) OR TI(reason*) OR TI(contribut*) OR TI(cause*) OR TI(adherence) OR TI(behavior) OR TI(behaviour) OR TI(characteristic*) OR TI(self-efficacy) OR TI(compliance) OR TI(attitude*”) |
| #3 | (MH "Movement+") OR (MH "Health Behavior") OR (MH "Health Promotion") OR (MH "Sports+") OR AB(movement) OR TI(movement) OR AB(physical activit*) OR TI(physical activit*) OR AB(routine*) OR TI(routine*) OR AB(sport*) OR TI(sport*) OR AB(training) OR TI(training) OR AB(workout) OR TI(workout) OR AB(motion) OR TI(motion) OR AB(physical exercis*) OR TI(physical exercis*) OR AB(health monitoring) OR TI(health monitoring) OR AB(health behav*) OR TI(health behave*) OR AB(health promotion*) OR TI(health promotion) OR AB(motor activit*) OR TI(motor activit*) OR AB(health educat*) TI(health educat*) OR ((AB(behaviour*) OR TI(behaviour*”)) OR AB(behavior*) OR TI(behavior*) AND (AB(modification*” OR TI (“modification*”))) |
| #4 | #1 AND #2 AND #3 |
| #5 | Filters: from 2005 – 2020 |
